# Post-COVID-19 Vaccination CNS Magnetic Resonance Imaging Findings: A Systematic Review

**DOI:** 10.1155/2023/1570830

**Published:** 2023-06-29

**Authors:** Sadegh Ghaderi, Sana Mohammadi, Mehrsa Heidari, Seyedeh Shadi Sharif Jalali, Mahdi Mohammadi

**Affiliations:** ^1^Department of Neuroscience and Addiction Studies, School of Advanced Technologies in Medicine, Tehran University of Medical Sciences, Tehran, Iran; ^2^Department of Medical Sciences, School of Medicine, Iran University of Medical Sciences, Tehran, Iran; ^3^Department of Medical Science, School of Medicine, Ahvaz Jundishapur University of Medical Sciences, Ahvaz, Iran; ^4^Department of Medical Physics, School of Medicine, Kermanshah University of Medical Sciences, Kermanshah, Iran; ^5^Department of Medical Physics and Biomedical Engineering, School of Medicine, Tehran University of Medical Sciences, Tehran, Iran

## Abstract

**Objective:**

This systematic review aims to synthesize and analyze the available literature on central nervous system (CNS) magnetic resonance imaging (MRI) findings in individuals who have received COVID-19 vaccinations. Our objective is to enhance understanding of potential neurological side effects, inform clinical practice, and guide future research on the neurological implications of COVID-19 vaccination.

**Methods:**

In this systematic review, we conducted a comprehensive search in PubMed, Scopus, and Web of Science from January 2020 to April 2023, using terms related to COVID-19 vaccination and CNS MRI findings. We evaluated the quality of the study, extracted relevant data, and included 89 eligible studies that covered various vaccines, demographics of patients, symptoms, and MRI findings to provide a thorough understanding of SARS-CoV-2 vaccination-related CNS problems.

**Results:**

We investigated CNS MRI findings following COVID-19 vaccination across various vaccine types. Common diseases associated with post-vaccination CNS MRI findings included cerebral venous sinus thrombosis (CVST), vaccine-induced immune thrombotic thrombocytopenia (VITT), acute disseminated encephalomyelitis (ADEM), acute myelitis, autoimmune encephalitis (AE), and others. Patients presented with diverse onset symptoms and neurological manifestations. Abnormalities identified in CNS MRI findings included white matter (WM) hyperintensity. Our analysis offers a comprehensive overview of the current literature on post-vaccination CNS MRI findings. *Discussion*. We highlight a range of post-COVID-19 vaccination CNS MRI findings, including CVST, with a higher incidence in individuals receiving the ChAdOx1 (AstraZeneca) vaccine. Other notable observations include cases of ADEM, myelitis or transverse myelitis (TM), Guillain–Barré syndrome (GBS), and acute encephalopathy following COVID-19 vaccination. The incidence of these neurological complications is extremely rare, and the benefits of vaccination outweigh the risks. The reviewed studies were primarily case reports or case series, and thus large-scale epidemiological studies and controlled clinical trials are needed to better understand the underlying mechanisms and risk factors associated with these neurological complications following COVID-19 vaccination.

## 1. Introduction

The coronavirus disease 2019 (COVID-19) pandemic, caused by the coronavirus 2 of the severe acute respiratory syndrome (SARS-CoV-2), has had a profound impact on global public health and resulted in significant morbidity and mortality rates [1–5]. The disease, initially recognized for its respiratory manifestations, has demonstrated multi-organ involvement, including the central nervous system (CNS). In order to mitigate the pandemic's devastating effects, vaccination programs have been implemented worldwide, aiming to reduce morbidity and mortality and ultimately achieve herd immunity. Various SARS-CoV-2 vaccines have been developed, with clinical trials demonstrating their efficacy in preventing severe COVID-19 infection [3, 6, 7].

The implementation of vaccination programs is crucial for controlling the spread of COVID-19 and protecting public health. Understanding potential side effects related to vaccines is essential for both healthcare providers and the general population. Common adverse effects following vaccination include pain, swelling, localized erythema at the injection site, fever, chills, fatigue, myalgia, muscle pain, vomiting, arthralgia, and lymphadenopathy [2, 6, 8–10]. In addition, some individuals have reported mild neurological symptoms, such as headaches, dizziness, myalgia, muscle spasticity, and paresthesia. A limited number of case reports have documented more serious side effects, including generalized seizures, Guillain–Barré syndrome (GBS), and transverse myelitis (TM). Other neurological symptoms, such as facial nerve palsy, acute disseminated encephalomyelitis, and stroke, have been reported [6, 8, 11, 12].

Magnetic resonance imaging (MRI) is one of the most crucial, extensively utilized, and powerful imaging modalities that offer numerous advantages, such as high spatial resolution, unrestricted tissue depth penetration, multi-planar imaging, and soft tissue functionality [13–15]. MRI has been used in various medical aspects and biomedical applications, including diagnosis, therapy, follow-up, and treatment assessment; cancer imaging; inflammation detection and perfusion imaging; and targeted MRI-guided drug delivery, among others. In light of the increasing number of COVID-19 vaccinations administered worldwide and the potential neurological side effects, understanding and characterizing the CNS MRI findings post-vaccination is crucial [6, 16–19].

In this study, our objective was to systematically review the existing literature on CNS MRI findings after COVID-19 vaccination. By providing a comprehensive analysis of the current evidence, we seek to contribute to the understanding of the potential neurological implications of COVID-19 vaccination and inform clinical practice and future research.

## 2. Methods

### 2.1. Search Strategy and Inclusion and Exclusion Criteria

The Preferred Reporting Items for Systematic Reviews and Meta-Analyses (PRISMA) flowchart is a structured and transparent approach to identifying, screening, and selecting studies for inclusion in a systematic review or meta-analysis [20, 21]. We designed a comprehensive search strategy to identify studies that examined post-COVID-19 vaccination CNS MRI findings. Our search was performed in three databases: PubMed, Scopus, and Web of Science, and covered the period from January 2020 to April 2023. The main search terms were as follows: ((COVID-19) AND (magnetic resonance imaging)) AND (vaccination) AND (Brain) and ((COVID-19) AND (magnetic resonance imaging)) AND (Vaccination) AND (spinal cord). Two reviewers (SG and SM) independently screened the search results. Primary research publications that present the implications of post-COVID-19 vaccination CNS MRI findings were included according to the inclusion criteria. Studies were excluded if they did not involve MRI imaging. Articles in all languages were considered for inclusion.

In addition to the database search, we reviewed existing reviews and checked their reference lists to identify any relevant studies that might have been missed during the initial search. Through this comprehensive search and selection process, we aimed to ensure that only relevant studies were included in our analysis.

### 2.2. Quality Assessment and Data Extraction

After the initial screening, articles were identified for further analysis based on the inclusion and exclusion criteria. To assess the quality of the selected studies, both reviewers independently evaluated the methodologies and results presented in each article. Discrepancies between the reviewers were resolved through discussion and consensus. Data extraction was performed using a standardized data extraction form. The extracted information included first author (year), vaccine, past medical history, gender/age, diseases, symptoms, and MRI findings. The data were then inserted into Table 1 for further analysis.

### 2.3. Characteristics of Eligible Studies

**Figure 1:**
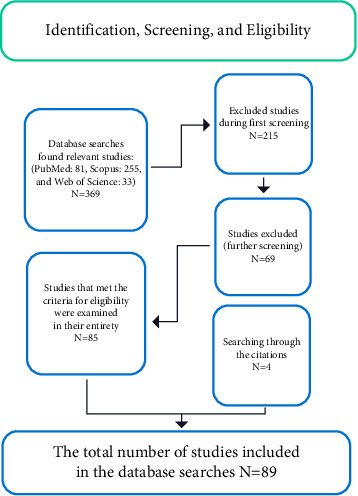
PRISMA flowchart: identification, screening, and eligibility.

The 89 eligible studies included in our systematic review investigated the implications of post-COVID-19 vaccination CNS MRI findings (Figure 1). To be included, studies needed to investigate the MRI modality simultaneously in conjunction with post-COVID-19 vaccination. The selected studies covered a diverse range of vaccines, patient demographics, clinical symptoms, and MRI findings, providing a comprehensive overview of the potential adverse effects and mechanisms of SARS-CoV-2 vaccination-related problems in the CNS.

## 3. Results

### 3.1. Overview of Results

Our systematic review included 89 eligible studies, which consisted of case reports, case series, and an original article by Nistri et al. [64]. Studies investigated the CNS MRI findings following COVID-19 vaccination, covering various vaccine types, including Pfizer-BioNTech, Ad26.COV2.S (Jcovden), ChAdOx1 (AstraZeneca), Johnson & Johnson, BIBP-CorV (Sinopharm), Sinovac-CoronaVac, mRNA-1273 (Moderna), and others. The medical history of patients in the included studies most commonly involved hypertension (HTN) and diabetes mellitus (DM). The gender distribution was approximately equal between men and women, and the age range of the patients spanned from 12 to 88 years.

The studies examined a variety of diseases associated with post-vaccination CNS MRI findings, with the most common being cerebral venous sinus thrombosis (CVST), vaccine-induced immune thrombotic thrombocytopenia (VITT), acute disseminated encephalomyelitis (ADEM), acute myelitis, and autoimmune encephalitis (AE), among others. Patients in the included studies presented with a range of onset symptoms, which prompted them to seek diagnostic and treatment services. The post-vaccination CNS MRI findings were diverse and encompassed a wide array of neurological manifestations.

Regarding the findings of CNS MRI, the studies reported a range of abnormalities, such as white matter (WM) hyperintensity. In summary, our systematic review provides a comprehensive analysis of the existing literature on the CNS MRI findings after COVID-19 vaccination.

## 4. Discussion

MRI is commonly used as a diagnostic approach for both short-term and long-term neurological diseases that may be caused by vaccine injection because of its excellent soft tissue contrast resolution. The primary objective of this systematic review was to provide a comprehensive analysis of the literature on CNS MRI findings following COVID-19 vaccination. Our search strategy identified 89 eligible studies, which revealed a wide range of vaccines, patient demographics, clinical symptoms, and MRI findings. The following discussion will focus on the key findings and implications of these studies.

A noteworthy observation from our analysis is the occurrence of CVST following COVID-19 vaccination, which was reported in several studies [22–33]. The incidence of CVST was found to be higher in patients who received the ChAdOx1 vaccine (AstraZeneca) compared to those who received the mRNA-based vaccines (Pfizer-BioNTech and Moderna) or Ad26.COV2.S (Janssen) vaccine. This observation is in line with previous reports which have suggested an association between the ChAdOx1 vaccine and an increased risk of CVST [110].

The clinical presentation of CVST varied among the included studies, with the most common symptoms being headache, fever, and vomiting [22–33]. In some cases, more severe neurological manifestations such as altered mental status (AMS), visual disturbances, and seizures were reported [31–33]. MRI findings in patients with CVST included hyperintensity on T2-weighted (T2-w) and fluid-attenuated inversion recovery (FLAIR) images, as well as restricted diffusion on diffusion-weighted imaging (DWI) sequences, indicating acute ischemic lesions [22, 23, 25, 26, 31].

It is important to note that the incidence of CVST following COVID-19 vaccination is still relatively low, and the benefits of vaccination in preventing severe COVID-19 infection and its complications far outweigh the risks associated with rare adverse events such as CVST [111]. Clinicians should remain vigilant for the early signs and symptoms of CVST in patients who have recently received a COVID-19 vaccine, as prompt diagnosis and treatment are essential for optimal patient outcomes [112].

CVST and VITT with CVST varied in symptoms, ranging from headache and abdominal pain to more severe manifestations such as visual loss, seizures, or encephalopathy [34, 35]. As with other cases of CVST post-vaccination, MRI and MRV were able to detect the thromboses as well as secondary complications like venous infarction or hemorrhage. These findings further highlight the need for clinicians to maintain a high index of suspicion of CVST and associated conditions in patients who present with neurological symptoms after COVID-19 vaccination, especially with the ChAdOx1 vaccine. Our analysis demonstrates that CVST and VITT with secondary CVST may be potential complications of COVID-19 vaccination requiring vigilance and early recognition. In particular, the risk of CVST appears to be higher following the ChAdOx1 vaccine compared to other vaccine platforms [34, 35].

Some of the cases identified in this review were associated with VITT following the administration of the ChAdOx1 (AstraZeneca) and the Johnson & Johnson vaccines [36, 37, 39–42]. A case of VITT was reported after vaccination with Pfizer-BioNTech [38]. The patients presented with a range of symptoms, including headaches, seizures, neurological deficits, and limb swelling. Thrombotic events, such as CVST, were the most common complications [36, 37, 39–42]. CVST has been reported as a rare adverse event following COVID-19 vaccination, particularly with the ChAdOx1 (AstraZeneca) vaccine. The MRI findings included thrombotic occlusions of various venous sinuses, acute intracerebral hematomas, and ischemic infarctions [36–42]. Early diagnosis is crucial for the appropriate management of these patients, which may involve anticoagulation therapy, intravenous immunoglobulin, or corticosteroids [92]. However, healthcare professionals should remain vigilant for potential neurological symptoms in patients following COVID-19 vaccination, particularly those who have received ChAdOx1 (AstraZeneca) and Johnson & Johnson vaccines.

A notable observation in our analysis was the prevalence of ADEM as a reported neurological complication after COVID-19 vaccination. ADEM is an autoimmune inflammatory demyelinating disease of the CNS that usually occurs after viral infection or vaccination [43, 45]. In our review, several ADEM cases were reported following vaccination with different COVID-19 vaccines such as mRNA-1273 (Moderna) [43], Pfizer-BioNTech [44, 47, 48], ChAdOx1 (AstraZeneca) [35, 45, 50], and BIBP-CorV (Sinopharm) [46]. The onset of symptoms varied from a few days to several weeks after vaccination, and the MRI findings showed multiple hyperintense lesions in different brain regions and spinal cord on T2-w and FLAIR sequences [43–48, 50, 103].

Another interesting finding in our review was the presence of MOG antibody-associated disorder (MOGAD) in two cases following COVID-19 vaccination [50, 51]. MOGAD is a rare autoimmune demyelinating condition that affects the optic nerves, spinal cord, and brain. In both cases, patients developed neurological symptoms after receiving a COVID-19 vaccine, and their MRI findings revealed hyperintense lesions in different brain regions with MOG-antibody positivity [50, 51]. This raises the question of whether COVID-19 vaccination can trigger MOGAD in susceptible individuals, which warrants further investigation.

Some of the included studies reported cases of myelitis or TM following COVID-19 vaccination, suggesting a possible association between vaccination and the development of these conditions. For instance, Dams et al. [52] reported a case of MOG-antibody-associated longitudinally extensive transverse myelitis (LTEM) following the ChAdOx1 (AstraZeneca) vaccine. Similar findings were reported by Sepahvand et al. [53], Miyaue et al. [54], Esechie et al. [55], and Erdem et al. [56] in patients who received the BIBP-CorV (Sinopharm), Pfizer-BioNTech, mRNA-1273 (Moderna), and Sinovac-CoronaVac vaccines, respectively. These cases suggest that various COVID-19 vaccines may be associated with the development of myelitis or TM, warranting further investigation to better understand the underlying mechanisms and potential risk factors. ATM was also reported in several cases following COVID-19 vaccination. Hirose et al. [57] and Eom et al. [58] described cases of ATM following mRNA-1273 (Moderna) and Pfizer-BioNTech vaccination, respectively. Alabkal et al. [59] reported a case of TM following the Pfizer-BioNTech vaccine, while Chen et al. [60] described a concurrent vasculitis in vertebral bodies and partial TM in a patient whose vaccine type was not specified. These cases add further evidence to the potential association between COVID-19 vaccination and the development of ATM or TM.

Neuromyelitis optica spectrum disorder (NMOSD) was reported in two cases following COVID-19 vaccination. Chen et al. [62] described a case of NMOSD in a middle-aged female following an inactivated virus vaccine, while Umezawa et al. [63] reported a case of NMOSD in a patient with a history of GBS who received the Pfizer-BioNTech vaccine. These cases suggest that COVID-19 vaccination might be associated with the development of NMOSD in certain individuals, particularly those with a history of autoimmune or demyelinating disorders. A rare case of vaccine-associated herpes simplex encephalitis was reported by Chen et al. [61] in a patient who received the Pfizer-BioNTech vaccine. This finding highlights the importance of considering the potential for atypical neurological complications following COVID-19 vaccination and underscores the need for further research to better understand the possible underlying mechanisms.

Some studies included demyelinating diseases, GBS, and anti-NMDAR encephalitis. Demyelinating diseases, particularly multiple sclerosis (MS) and clinically isolated demyelinating syndrome, were observed in several cases [65, 66]. The majority of these patients had a history of MS or other demyelinating disorders, suggesting that the COVID-19 vaccination might have induced an exacerbation of the pre-existing condition. Interestingly, the vaccines involved in these cases were mRNA-based (Moderna and Pfizer-BioNTech) and inactivated virus-based (Sinopharm). This observation indicates that the potential of inducing or exacerbating demyelinating diseases might not be limited to a specific vaccine type.

GBS was another neurological condition identified in post-vaccination CNS MRI findings [68–72]. GBS is an immune-mediated disorder characterized by rapidly developing muscle weakness and, in some cases, paralysis. The cases involved patients who received either the ChAdOx1 (AstraZeneca), Pfizer-BioNTech, or Ad26.COV2.S (Janssen) vaccines. These results suggest that GBS might be a potential neurological complication of COVID-19 vaccination, although the incidence remains rare. It is worth noting that GBS has been previously associated with other vaccines, including influenza and other viral vaccines [113, 114].

One case reported by Etemadifar et al. [65] described a patient who developed anti-NMDAR encephalitis, an autoimmune disorder, after receiving the Sinopharm BIBP-CorV vaccine. The patient presented with various symptoms, including myalgia, ataxia, and dizziness, and the MRI findings revealed multiple new plaques in the periventricular, juxtacortical, and cortical areas. This case highlights the potential for post-vaccination neurological complications related to autoimmunity.

Kobayashi et al. [73] reported a case of AE following Pfizer-BioNTech vaccination, presenting with acute-onset diplopia and a lesion on the dorsal pons across the midline on brain MRI. Similarly, Rastogi et al. [74] observed acute encephalopathy in a patient who received ChAdOx1 (AstraZeneca) and mRNA-1273 (Moderna) vaccines, with multiple focal, poorly defined regions of contrast enhancement in the cerebral cortex, deep grey matter, brainstem, and cerebellum on brain MRI. These cases suggest that acute encephalopathy may be a potential neurological manifestation of COVID-19 vaccination.

Vences et al. [75, 76] reported two cases of AE following COVID-19 vaccination. In the first case, a patient with DM and HTN developed neurological symptoms after receiving the Pfizer-BioNTech vaccine. MRI revealed hyperintense lesions in various brain regions that evolved between the first and second doses of the vaccine. In the second case, a patient developed neurological symptoms after receiving the BIBP-CorV (Sinopharm) vaccine. MRI showed small hyperintense non-specific demyelinating lesions in several brain regions, with some lesions evolving over time. These findings may suggest a possible link between COVID-19 vaccination and the development of acute encephalopathy or demyelinating lesions in some individuals. Zhang et al. [77] described a case of AE following BIBP-CorV (Sinopharm) vaccination, with abnormal signals in the splenium of the corpus callosum on brain MRI. This case adds to the growing body of evidence suggesting that COVID-19 vaccination may be associated with various CNS manifestations, although further studies are needed to confirm this association and to understand the underlying mechanisms.

Huang and Huang [78] reported a case of autoimmune encephalopathy following ChAdOx1 (AstraZeneca) vaccination. The patient presented with acute-onset amnesia, language disturbance, and seizure, and brain MRI revealed a subacute infarction at the right internal capsule and irregular vascular contour. This case highlights the potential for autoimmune encephalopathy to occur following COVID-19 vaccination.

Some reviewed studies consistently reported the presence of cytotoxic lesions of the corpus callosum (CLOCCs) following vaccination. Youn and Yang [79] reported a case of a 22-year-old male presenting with febrile sensation and headache around the eyes and forehead after receiving the Pfizer-BioNTech vaccine. The MRI findings showed restricted diffusion in the corpus callosum, characterized by low ADC values and lack of contrast-mediated enhancement. Similar findings were observed by Poussaint et al. [80], who described a 12-year-old male with Lyme disease experiencing severe headache and visual hallucinations following vaccination. In this case, the MRI revealed T2-w prolongation and lower diffusivity in the splenium of the corpus callosum.

Procaccini et al. [81] described a 51-year-old woman who presented with fever, weakness, headache, palpitations, malaise, and AMS after receiving the Pfizer-BioNTech vaccine. MRI findings revealed a single oval-shaped lesion located in the splenium of the corpus callosum with hyperintense signal on T2-w and FLAIR, as well as restricted diffusivity on DWI. Interestingly, this study also provided follow-up MRI data, which showed complete resolution of the lesion in the splenium after 17 days and the persistence of small round-shaped WM hyperintensities on T2-w and FLAIR after 67 days. Lastly, Ohara et al. [82] reported two cases of CLOCCs in patients with different medical histories after receiving the Pfizer-BioNTech vaccine. Both patients exhibited similar MRI features, including restricted diffusion in the splenium with low ADC values and high signal intensity lesions on FLAIR images. All the CLOCC cases reviewed here involved the Pfizer-BioNTech vaccine, and further research is needed to determine whether similar MRI findings occur following other COVID-19 vaccines.

CNS-related events, such as ischemic stroke, spinal cord ischemia, acute hemorrhagic encephalomyelitis (AHEM), intracerebral bleeding, and others, were reported. Blauenfeldt et al. [83] described a case of acute ischemic stroke in a 60-year-old woman with a history of Hashimoto's thyroiditis and HTN following the ChAdOx1 (AstraZeneca) vaccine. Similarly, Elaidouni et al. [84] reported a case of acute ischemic stroke in a 36-year-old man after receiving the BIBP-CorV (Sinopharm) vaccine. In both cases, the patients presented with neurological symptoms, such as headache, numbness, and weakness, and their brain MRI findings confirmed the presence of ischemic stroke.

Cases of prolonged migraine aura resembling ischemic stroke have also been reported following the Sinovac-CoronaVac vaccine [85, 86]. Both patients were young females (24 years old) with neurological symptoms, including visual disturbance, tingling, and numbness. Brain MRA findings in both cases revealed mild irregularities of the intracranial arteries. Yoshida et al. [87] reported a case of cardioembolic stroke in an 83-year-old woman with atrial fibrillation and osteoarthritis following the Pfizer-BioNTech vaccine. The patient experienced left hemiplegia and left hemispatial neglect, and brain MRI findings showed acute infarction in the left insular cortex and corona radiates, as well as occlusion of the left middle cerebral artery (MCA). After the second dose of the vaccine, the patient developed another ischemic stroke, with acute infarction in the right insular cortex, caudate, and corona radiate, and occlusion of the right MCA. Spinal cord ischemia was reported by Fotiadou et al. [88] in a 59-year-old man following the Pfizer-BioNTech vaccine. The patient presented with right lower limb weakness, abdominal pain, and paraplegia. Spine MRI findings revealed T2-w hyperintensities extending from the T6 level to the conus medullaris and bilaterally symmetric circular high signal foci in the anterior horn cells of the spinal cord.

AHEM has been described in three cases following the ChAdOx1 (AstraZeneca) vaccine [89]. The patients exhibited various neurological symptoms, including fever, headache, seizures, back pain, weakness, nausea, and dizziness. Brain and spine MRI findings in these cases showed FLAIR hyperintense lesions with hemorrhagic involvement in different regions of the CNS, such as the basal ganglia, WM, and spinal cord. Kits et al. [90] reported a case of AHEM in a 53-year-old man with rheumatoid arthritis following the Pfizer-BioNTech vaccine. The patient presented with confusion, unconsciousness, agitation, and snoring. Brain MRI findings showed multiple cortical and subcortical lesions with high T2-w and FLAIR signal and ubiquitous petechial hemorrhages. Repeated MRI demonstrated the development of widespread lesions in various CNS regions and cortical laminar necrosis. Lastly, Finsterer and Korn [91] reported a case of intracerebral bleeding in a 52-year-old man with a history of myocardial infarction, hypertension, hyperlipidemia (HLP), and nephrolithiasis following an mRNA-based SARS-CoV-2 vaccine. The patient experienced sudden-onset difficulties with reading and speaking (aphasia), and cerebral MRI findings showed intracerebral bleeding in the left temporal lobe.

We observed a wide range of neurological manifestations following COVID-19 vaccination in the included studies. These manifestations included optic neuropathy [93], inflammatory demyelinating polyneuropathy [94], acute small fiber neuropathy [95], unilateral multiple cranial neuropathy [96], cranial nerve palsies [97], Bell's palsy [98], ischemic stroke [99], and polyneuritis cranialis [100]. The onset symptoms varied across studies but commonly involved pain, sensory changes, and motor deficits. In the majority of the cases, the reported MRI findings were consistent with the clinical presentation of the patients. For example, Elnahry et al. [93] reported significant enhancement of the left optic nerve in a patient with optic neuropathy, while Bagella et al. [94] found enhancement of the facial nerves and cauda equina in a patient with inflammatory demyelinating polyneuropathy. Similarly, other studies reported abnormal enhancement of cranial nerves in patients presenting with cranial neuropathies [96–98, 100] or ischemic lesions in a patient with ischemic stroke [99]. In some cases, the MRI findings were less specific or incidental, such as the multiple perineural cysts found in a patient with acute small fiber neuropathy [95].

Finally, in recent literature, there have been several case reports describing concerns about potential neurological complications [101–105, 107–109]. These findings include cerebral infarctions [101], demyelinating diseases [102–104], hemorrhagic events [105], and infections [107], among others. While these case reports suggest a potential association between COVID-19 vaccination and neurological complications, it is important to note that the rarity of these events and the presence of confounding factors, such as pre-existing medical conditions, necessitate further investigation to establish causality. The development of multi-systemic inflammatory syndromes and the exacerbation of hereditary neuropathies after COVID-19 vaccination have also been reported in the literature [107, 109]. Recent case reports have documented a variety of CNS MRI findings following COVID-19 vaccination, raising questions about potential neurological complications [101–105, 107–109]. These findings encompass cerebrovascular events, demyelinating processes, hemorrhagic events, infections, and inflammatory processes, among others. While these cases suggest a potential association between COVID-19 vaccination and neurological complications, it is crucial to consider that the rarity of these events and the presence of confounding factors, such as pre-existing medical conditions, require additional research to establish causality.

In sum, one of the important frameworks is the World Health Organization (WHO) causality assessment framework for adverse events following immunization (AEFI). The WHO has developed protocols and algorithms to determine the causality relationship between vaccine administration and AEFI in a standardized manner. These protocols consider factors such as the temporal relationship between vaccination and the event, alternative explanations, past medical history, laboratory tests, and outcomes upon rechallenge. By following the WHO framework, researchers can evaluate the likelihood of a true causal association between vaccination and AEFI on a six-point scale from “very likely” to “unlikely.” Implementing the WHO causality assessment framework would strengthen future studies examining post-COVID-19 vaccination CNS MRI findings. First, it allows for objective and standardized determinations of causality that can be compared across studies. Second, it facilitates the detection of possible safety signals by distinguishing events that are very likely or likely due to vaccination from those that are indeterminate or unlikely to be causally related. Third, for events categorized as very likely or likely due to vaccination, the WHO framework guides the recommendation of further epidemiological study to confirm or refute the signal [115, 116].

However, limited data and lack of control groups in the included case reports and case series in this review highlight the challenges of conducting a full WHO causality assessment. Large, rigorous epidemiological studies on events that appear potentially related to vaccination based on existing cases are needed to enable comprehensive causality determinations. While case reports play an important role in initial signal detection, controlled observational studies and randomized trials remain the gold standard evidence required for policy decision making regarding vaccine safety.

## 5. Conclusion

These findings highlight the importance of MRI as a diagnostic tool in identifying potential neurological complications following COVID-19 vaccination. It is important to note that the incidence of these neurological complications is extremely rare and the overall benefits of COVID-19 vaccination in preventing severe illness and death far outweigh the risks. It is important to note that the reviewed studies were case reports or case series, and the number of reported cases with post-vaccination CNS MRI findings is relatively small compared to the millions of COVID-19 vaccine doses administered worldwide. Therefore, the incidence of these neurological complications following COVID-19 vaccination remains unclear. Comprehensive and systematic investigations, including large-scale epidemiological studies and controlled clinical trials, are crucial to understanding the underlying mechanisms and risk factors associated with these neurological complications and informing public health recommendations.

## Figures and Tables

**Table 1:** Post-COVID-19 vaccination CNS MRI findings.

Author (year)	Vaccine	Past medical history	Gender/age	Diseases	Onset symptoms	MRI findings
D'Agostino et al. (2021) [22]	ChAdOx1 (AstraZeneca)	NA	F/54	CVST	Left side signs	(i) Acute basilar thrombosis associated with the superior sagittal sinus thrombosis with the delineation of hyperacute ischemic lesions in the vascular territory of the right posterior cerebral artery and of the perforating pontine branches
						(ii) Acute ischemic lesion with restricted diffusion involving the pons, mesencephalon, the right superior cerebellar hemisphere with the vermis, and the right posterior temporal lobe

Jamme et al. (2021) [23]	ChAdOx1 (AstraZeneca)	HTN	F/69	CVST	Headache associated with behavioral symptoms unconscious	(i) Bilateral frontal hemorrhage with brain herniation aggravating a cerebral venous thrombosis of the left internal jugular vein, sigmoid sinus, and superior sagittal sinus

Dutta et al. (2021) [24]	ChAdOx1 (AstraZeneca)	NA	M/51	CVST	Headache and vomiting	(i) Extensive venous collaterals in the superior sagittal sinus and transverse sinus observed in MRV

Ikenberg et al. (2021) [25]	ChAdOx1 (AstraZeneca)	NA	F/30	CVST	Mild myalgia, a holocephalic headache, and chills	(i) CVST of the left transverse and sigmoidal sinus with a left-temporal and left-cerebellar intracerebral hemorrhage

Wang et al. (2021) [26]	ChAdOx1 (AstraZeneca)	NA	F/41	CVST	Fever, headache, pain, and swelling erythema on bilateral palms	(i) The focal T1-w intermediate-signal-intensity and T2-w hyperintensity lesion within the posterior superior sagittal sinus
						(ii) Filling defect within the posterior superior sagittal sinus

Syed et al. (2021) [27]	mRNA-1273 (Moderna)	DM	M/45	CVST	Headaches and neck pain	(i) Intracranial hemorrhage within the anterior left frontal lobe, bifrontal subarachnoid hemorrhage, and anterior superior sagittal sinus thrombosis

Yamaguchi et al. (2021) [28]	Pfizer-BioNTech	Hyperuricemia	M/61	CVST	Headache and fever	(i) Thrombosis in the superior sagittal sinus and the right transverse sinus

Braun et al. (2021) [29]	ChAdOx1 (AstraZeneca)	NA	M/21	CVST	Fever and headache	(i) Thrombosis of the superior sagittal sinus
						(ii) Left-sided thrombosis in the internal jugular vein, sigmoid sinus, and lateral portion of the transverse sinus. Ischemia detected in the left cerebellar and the right high parietal and parieto-occipital cortex.

Finsterer and Nicset (2021) [30]	Pfizer-BioNTech	NA	M/33	CVST	Headache in the left temporal region with nausea and vomiting	(i) Venous sinus thrombosis of the left transverse and sigmoid sinuses

Nicolardi et al. (2022) [31]	Pfizer-BioNTech	NA	M/56	CVST	Severe asthenia, headache and acute confusional state	(i) T2-w hyperintensity areas in both thalami
						(ii) FLAIR: ischemic phenomena in thalami, left hippocampal, para hippocampal regions and central portion of splenium of the corpus callosum and hemorrhagic lesion in left para hippocampal region
						(iii) SWI: heavy metal deposits in the aforementioned regions
						(iv) PWI: low levels of CBF and CBV in affected areas

Qureshi et al. (2022) [32]	Pfizer-BioNTech	HTN, DM, CKD, essential tremors, anxiety, and depression	M/80	CVST	Headache, tinnitus, and visual disturbance	(i) The MRV indicates a lack of blood flow in the left transverse sinus and left jugular vein within the cerebral venous sinuses

Elfil et al. [33]	Ad26.COV2.S (Jcovden)	NA	M/28	CVST	Headache, blurry vision, diplopia, and photophobia	(i) Filling defects within the superior sagittal sinus as well as the right transverse sinus extending into the right jugular vein

Bonato et al. (2021) [34]	ChAdOx1 (AstraZeneca)	NA	F/26	CVST and VITT	Headache, weakness, and visual disturbances	(i) An extensive venous infarction with hemorrhagic transformation in the right parietal and left frontoparietal lobes
						(ii) Multi-focal venous thrombosis, with bilateral blockage of the parietal cortical veins, straight sinus, vein of Galen, internal cerebral veins, and inferior sagittal sinus

Graf et al. (2021) [35]	ChAdOx1 (AstraZeneca)	NA	M/29	VITT with CVST	Headache, abdominal pain, abdominal cramps, and hematemesis	(i) Thrombosis of the left transverse and sigmoid sinuses, as well as the left proximal jugular vein

Wolf et al. (2021) [36]	ChAdOx1 (AstraZeneca)	NA	(1) F/22	VITT	(1) Frontally accentuated headaches and a self-limited generalized epileptic seizure	(1) Blood in the subarachnoid space adjacent to the falx cerebri on both sides. Thrombotic occlusion of the superior sagittal sinus, the left-hand transverse sinus, and the sigmoid sinus.
			(2) F/46		(2) Mild aphasia and hemianopia to the right	(2) Thrombotic occlusion of the superior sagittal sinus and the left-hand transverse sinus and sigmoid sinus. An acute intracerebral hematoma in the left occipital lobe.
			(3) F/36		(3) Headache and somnolence with right-hand hemiparesis	(3) Thrombotic occlusion of the straight sinus and a non-occlusive thrombus in the superior sagittal sinus. Congestive edema of both thalami with more pronounced on the left side.

Hsiao et al. (2022) [37]	ChAdOx1 (AstraZeneca)	NA	F/40	VITT	Chest pain, headache, and abdominal pain	(i) MRV shows CVST

Goh Cy et al. (2022) [38]	Pfizer-BioNTech	NA	M/76	VITT	Left upper limb swelling	(i) Acute left MCA thrombosis with infarction

Gattringer et al. (2022) [39]	ChAdOx1 (AstraZeneca)	NA	(1) F/39	VITT	(1) Severe holocephalic headache	(1) Left transverse sinus/sigmoid thrombosis but no brain parenchymal damage
			(2) F/24		(2) Severe persistent headache	(2) Thrombosis, cortical veins, and a small frontal juxtacortical hemorrhage found on the right side of the brain

Yahyavi-Firouz-Abadi and Naik (2022) [40]	Johnson & Johnson	NA	F/30	VITT	Worsening head and neck pain	(i) Large occlusive thrombus in the right transverse sinus extending to the right sigmoid sinus and jugular bulb

Jiang et al. (2022) [41]	ChAdOx1 (AstraZeneca)	Iron deficiency anemia	F/36	VITT	Headaches accompanied by left upper limb clumsiness	(i) T1-w hypointensity lesion at the right parietal lobe
						(ii) T2-w FLAIR hypointensity lesion
						(iii) SWI hypointensity “black dots” at the right parietal lobe
						(iv) An irregular contour of the right transverse sinus in MRV

Rodriguez et al. (2022) [42]	Johnson & Johnson	NA	F/37	VITT	Neurological deterioration and right hemiplegia	(i) Thrombosis of the major anterior part of the sagittal superior sinus with bilateral intraparenchymal hemorrhagic complications

Kania et al. (2021) [43]	mRNA-1273 (Moderna)	Atopic dermatitis and depression	F/19	ADEM	Severe headache, fever, back and neck pain with nausea and vomiting, and urinary retention	(i) T2-w FLAIR hyperintense lesions in both brain hemispheres, pons, the medulla oblongata, and cerebellum. Few of them were contrast‐enhanced lesions.
						(ii) T2‐w FLAIR widespread hyperintense area extended from medulla oblongata to T11 segment with overlapping few contrast‐enhancing lesions

Shimizu et al. (2021) [44]	Pfizer-BioNTech	DM and Alzheimer's disease	F/88	ADEM	Impaired consciousness, gaze, and evoked nystagmus	(i) DWI, T2-w, and FLAIR hyperintensity of the middle cerebellar peduncles
						(ii) CE MRI shows low signal intensity in the middle cerebellar peduncles

Nagaratnam et al. (2022) [45]	ChAdOx1 (AstraZeneca)	NA	F/36	ADEM	Bilateral optic neuritis, right-sided headache, photophobia, blurred vision, bilateral visual impairment, subjective color desaturation, painful eye movements, and fatigue	(i) T2-w FLAIR hyperintensity lesions involving the subcortical WM, posterior limb of bilateral internal capsules, pons, and left middle cerebellar peduncle. The abnormal signal and enhancement of both optic nerves but more prominent on the left.

Yazdanpanah et al. (2022) [46]	BIBP-CorV (Sinopharm)	NA	M/37	ADEM	Progressive weakness of 4 limbs, dysphagia, drooling, nausea, and vomiting. Symptoms of bilateral facial nerve paralysis.	(i) T2-w FLAIR hyperintensity foci within the left corticospinal tract in the left cerebral peduncle, right and left sides of the pons, and medulla
						(ii) Post-contrast T1-w: small enhancement in some of the lesions
						(iii) DWI: significant restriction at the level of the pons
						(iv) MRS: demyelination process by the presence of myoinositol and choline peaks

Poli et al. (2022) [47]	Pfizer-BioNTech	NA	M/65	ADEM, ocular myasthenia gravis, and autoimmune thyroiditis	Mild left-sided hemiparesis, contralateral dissociated sensory loss, dizziness, right-sided deafness, binocular horizontal diplopia, and ptosis of the right eye	(i) Multiple acute inflammatory contrast-enhancing periventricular and brainstem lesions with involvement of vestibulocerebellar tract and cochlear nuclei

Ahmad et al. (2022) [48]	Pfizer-BioNTech	HTN and anxiety	F/61	ADEM	Progressive generalized weakness and AMS	(i) Diffuse and near symmetric acute leukoencephalopathy process involving the deep WM extending downward through the brainstem into the cerebellar WM tracts

Garg et al. (2022) [17]	ChAdOx1 (AstraZeneca)	NA	F/67	ADEM	AMS, progressive loss of memory and cognitive ability, subtle personality changes, inability to concentrate, and lethargy	(i) Multiple nodular/oval T2-w FLAIR hyperintensities involving the deep and periventricular cerebral WM asymmetrically, corpus callosum, subcortical regions, bilateral middle cerebellar peduncles, bilateral cerebellar hemisphere, and left basal ganglia without any mass effect

Poli et al. (2023) [49]	Pfizer-BioNTech	ADEM with two fully remitted episodes 10-11 years ago	M/65	ADEM	Muscle weakness and numbness in all extremities	(i) Multiple acute inflammatory contrast-enhancing periventricular and right-sided brainstem lesions

Mumoli et al. (2021) [50]	ChAdOx1 (AstraZeneca)	Allergic asthma triggered by pollen	M/45	ADEM anti-MOG antibody-positive	Bilateral lower limb weakness, urinary retention, vertigo, fever, diffuse myalgia, and back pain	(i) Brain: FLAIR images showed large asymmetric lesions of WM, basal ganglia, and cortical GM with asymmetric involvement of thalami. Additionally, there is a tumefactive lesion present in the left frontal lobe
						(ii) Spine: STIR images showed large confluent lesions extending over multiple segments until conus

Matsumoto et al. (2022) [51]	mRNA-1273 (Moderna)	Intraductal papillary mucinous neoplasm and HTN	F/68	MOG antibody-associated disorder	Numbness on the right side of face	(i) T2-w FLAIR hyperintensity lesion on the right lateral pontine and trigeminal nerve, and a middle cerebellar peduncle lesion was seen

Dams et al. (2021) [52]	ChAdOx1 (AstraZeneca)	NA	M/59	MOG-antibody-associated LTEM	Paresthesia, gait disturbance, and urinary and rectal dysfunction	(i) On STIR images, it was observed that there is longitudinal myelopathy affecting the cervical, thoracic, and lumbar spinal cord, but without any contrast enhancement. The conus was also involved.

Sepahvand et al. (2021) [53]	BIBP-CorV (Sinopharm)	DM, HTN, and ischemic heart disease	M/71	LTEM	Hemiparesis, left paresthesia in both hands, and urinary retention	(i) Longitudinally extensive T2-w hyperintense lesion without gadolinium enhancement from cervico-medullary junction to C3 level predominantly on the left side of the spinal cord

Miyaue et al. (2021) [54]	Pfizer-BioNTech	HTN, HLP, and prostate cancer	M/75	LTEM	Ascending paresthesia, lower back pain, reduced sensation during urination and defecation, and severe weakness in both legs	(i) T2-w longitudinally extensive hyperintense lesion from the lower thoracic to lumbar spine was seen

Esechie et al. (2022) [55]	mRNA-1273 (Moderna)	Metastatic small-cell lung cancer, HTN, BPH, and chronic back pain with placement of a spinal stimulator	M/58	LTEM	Paralysis of the lower extremity and sensory loss from chest down with overflow incontinence	(i) Enhancing lesions from C7-T7 concerning for LTEM

Erdem et al. (2021) [56]	Sinovac-CoronaVac	NA	F/78	ATM	Tetraparesis, paresthesias of bilateral upper extremities, and urinary retention	(i) Extensive TM from the C1 to the T3 spinal cord segment

Hirose et al. (2021) [57]	mRNA-1273 (Moderna)	HTN, hyperuricemia, and alcoholic liver cirrhosis	M/70	ATM	Bilateral lower extremities hypoesthesia and mild paraparesis	(i) T2-w: multiple high-intense areas located at the T1/2 and T5/6 vertebral levels with weak gadolinium enhancement
						(ii) CE T1-w: focal weak gadolinium enhancement in the T2-w high-intense area

Eom et al. (2022) [58]	Pfizer-BioNTech	(1) HTN and DM	(1) M/71	ATM	(1) Bilateral hand weakness and numbness in fingers	(1) High signal intensity and multi-focal nodular enhancement with an ill-defined signal increase on T2-w from the C1 to C3 vertebrae
		(2) NA	(2) F/23		(2) Tingling sensation in both thighs, weakness of both legs, and urinary retention	(2) High signal intensity lesion without contrast enhancement at the anterior portion of the conus medullaris on T2-w

Alabkal et al. (2021) [59]	Pfizer-BioNTech	Pancreatitis and recurrent urinary tract infections	F/26	TM	Saddle anesthesia and bilateral paresthesias, numbness, and intermittent allodynia ascending the plantar aspects of the feet up the posterior legs	(i) T2-w hyperintense and diffusely enhancing lesion at T5, in keeping with TM
						(ii) Three non-specific small patchy T2-w hyperintense lesions within the supratentorial deep WM (right anterior frontal lobe, right frontal operculum, and left internal capsule) which were felt to be non-specific

Chen et al. (2022) [60]	NA	NA	M/39	Concurrent vasculitis in vertebral bodies and partial TM	Weakness of left lower limb and aberrant sensation of the left lower trunk and limb from T9 level to toes	(i) T2-w high signal in vertebral bodies of T3-T7 and at T7 spinal cord indicating an acute demyelinating lesion

Chen et al. (2023) [61]	(1) ChAdOx1 (AstraZeneca)	(1) DM, HTN, and HLP	(1) F/56	(1) Acute myelitis	(1) Numbness with burning sensation in right lower limb below inguinal region	(1) High T2-w from T10 to the upper L1 level with minimal faint enhancement. Old lacunar infarction of the bilateral corona radiata, bilateral basal ganglia, and corpus callosum.
	(2) Pfizer-BioNTech	(2) MDD	(2) M/64	(2) Vaccine-associated herpes simplex encephalitis	(2) Fever, AMS, nausea, and vomiting	(2) Hyperintensity in the bilateral cingulate gyrus

Chen et al. (2021) [62]	Inactivated virus vaccine	NA	F/middle-aged	NMOSD	Mild fever, vomiting, diarrhea, cough, dizziness, and unsteady walking	(i) Area postrema and bilateral hypothalamus lesions

Umezawa et al. (2022) [63]	Pfizer-BioNTech	GBS	F/52	NMOSD	Neck pain, weakness of the left arm and leg, numbness of the left hand, and impaired temperature sensation of the right leg	(i) T2-w and FLAIR hyperintense lesions reached from the C1 to C6 level. Gd-enhancement lesion from the C3 to C5 level and left lateral fasciculus
						(ii) T2-w and DIR hyperintense lesions in the area postrema and the obex of the medulla
Nistri et al. (2021) [64]	1–4. ChAdOx1(AstraZeneca)	1, 3, and 5–13. MS	(1) M/45	MS (relapse)	(1) Dysesthesia in both legs	(1) C3 lesion
	5 and 6. mRNA-1273 (Moderna)	2 and 4. NA	(2) F/48		(2) Visual acuity deficit from right eye	(2) Enhancing lesion in corpus callosum and multiple WM unenhanced lesions in periventricular areas and in the mesial occipital lobe
	7–16. Pfizer-BioNTech		(3) F/54		(3) Hypoesthesia below the T6 level	(3) New enhancing lesion in the thoracic cord
			(4) F/66		(4) Visual disturbance and postural instability on the right limbs	(4) Multiple hyperintense lesions in the supra and infratentorial WM, four of which are with contrast enhancement
			(5) F/42		(5) Slight weakness of the left upper limb	(5) New brain lesion with contrast enhancement
			(6) F/57		(6) Severe motor deficit in both legs	(6) New enhancing bulbar lesion
			(7) F/49		(7) Numbness on the left hand and left side of her head	(7) C3 lesion with contrast enhancement
			(8) M/39		(8) Paresthesia on the left leg	(8) New brain enhancing lesion
			(9) F/39		(9) Dysesthesia on the right hand and foot	(9) Contrast enhancing lesion in the mesencephalon
			(10) F/60		(10) Fatigue and numbness in both legs	(10) New enhancing brain lesion
			(11) F/30		(11) Language disturbance	(11) New enhancing brain lesion with conspicuous edema
			(12) F/58		(12) Headache, balance disturbances, urinary incontinence, difficulties in walking, and dysphagia	(12) Active lesion with ring enhancement in the left frontal WM
			(13) F/34		(13) Neck pain and hypoesthesia on the right arm	(13) Three new brain enhancing lesions, one of which is indicated by the arrow
			(14) F/35		(14) Paresthesia on the left side of the body	(14) Three new enhancing lesions in the left temporal lobe and one, indicated here, in the left centrum semiovale
			(15) F/54		(15) Right hemiparesis	(15) Two new ring-enhancing lesions localized in the WM adjacent to the left frontal horn and in the left middle periventricular region
			(16) F/37		(16) Weakness on the right limbs	(16) New enhancing lesions, one of which is tumefactive, localized in the WM of the left centrum semiovale

Etemadifar et al. (2022) [65]	BIBP-CorV (Sinopharm)	MS	F/50	Anti-NMDAR encephalitis, acute MS relapse, or a combination of both	Myalgia, precipitation, vomiting, leg weaknesses, ataxia, dizziness, fatigue, and mildly agitated with an ataxic gait	(i) Multiple new plaques in periventricular, juxtacortical, and cortical areas

Khayat‐Khoei et al. (2022) [66]	1, 2, and 6. mRNA-1273 (Moderna)	1, 3, and 6. MS	(1) F/35	1, 3, and 6. MS exacerbation	(1) Right arm dysmetria and gait ataxia	(1) T2-w hyperintense lesion in the right cerebellum enhanced with gadolinium
	3, 4, and 5. Pfizer-BioNTech	2, 4, and 5. NA	(2) F/26	2 and 5. Relapsing-remitting of MS	(2) Blurred vision and pain of right eye	(2) Multiple T2-w hyperintense periventricular, subcortical, posterior fossa, and spinal cord lesions (two of the lesions enhanced, one each in the brain and spinal cord)
		7. Isolated demyelinating syndrome	(3) F/24	(4) Neuromyelitis optica	(3) Vision changes and pain of the right eye	(3) Several new enhancing brain lesions without any optic MRI abnormalities
			(4) M/64	(7) Clinically isolated demyelinating syndrome	(4) Paresthesias, urinary retention, constipation, and balance/gait difficulty	(4) Longitudinally extensive high signal abnormalities with cord swelling in the thoracic spinal cord. Gadolinium-enhanced T1-w: patchy posterior lesion enhancement at T1/2-T5 and T9-T10/11
			(5) M/33		(5) Blurred vision of the left eye	(5) Multiple T2-w WM signal abnormalities in the corpus callosum with extension into the left frontal and parietal WM, suggestive of demyelination
			(6) F/44		(6) Ascending numbness and right-sided weakness	(6) Periventricular and juxtacortical T2-w lesions typical of MS. Spine MRI: a T2-w hyperintense thoracic spinal cord lesion.
			(7) F/48		(7) Pain of the right eye and balance/gait difficulty	(7) Periventricular and juxtacortical T2-w lesions and a new gadolinium-enhancing lesion in the centrum semiovale, adjacent to the falx in the left cerebral hemisphere
						(8) Three new T2-w hyperintense WM lesions

Czarnowska et al. (2023) [67]	Johnson & Johnson	HTN	M/33	MS	Right upper and lower extremities numbness	(i) Several demyelinating lesions, T1-w: subcortical region of the right frontal lobe lesion with gadolinium enhancement
						(ii) T2-w hyperintense lesion located in corpus callosum
						(iii) T2-w hyperintense acute lesion consistent with demyelination at C4/5 and one smaller lesion at the level of C3/C4, without enhancement

Patel et al. (2021) [68]	ChAdOx1 (AstraZeneca)	NA	M/37	GBS	Back pain and progressive ascending muscle weakness	(i) Both sides thickened cauda equina nerve rootlets, especially at the S1 level
						(ii) CE T1-w showed significant augmentation of both the conus medullaris and the ventral cauda equina nerve roots

Allen et al. (2021) [69]	ChAdOx1 (AstraZeneca)	(1) NA	(1) M/54	GBS	(1) Distal dysesthesia in the feet and hands and facial weakness	(1) CE subtle enhancement bilaterally in the distal facial nerves at the internal auditory canal. There was symmetric enhancement of the labyrinthine, tympanic, the labyrinthine, tympanic, and descending parts of the facial nerves showed symmetric enhancement
		(2) HTN	(2) M/55		(2) Bilateral thigh paresthesias, numbness in the sacral and lumbar regions, and development of facial diplegia	(2) CE enhancement of the facial nerve within the right internal auditory canal

Nishiguchi et al. (2021) [70]	Pfizer-BioNTech	DM	M/71	GBS	Headache and ocular pain	(i) Minor venous dilation of the middle cranial fossa

Dang and Bryson (2021) [71]	ChAdOx1 (AstraZeneca)	NA	M/63	GBS	Lower back pain, bilateral facial weakness, lower limb weakness, and paresthesia	(i) CE enhancement of the facial and oculomotor nerves bilaterally

Berrim et al. (2022) [72]	(1) Ad26.COV2.S (Jcovden)	—	(1) M/80	GBS	(1) Dysphonia, paresthesia, and quadriparesis	(1) Small vessel ischemic disease was noted
	(2) Pfizer-BioNTech		(2) M/62		(2) Peripheral facial weakness and ascending paralysis	(2) Diffuse enhancement of the left facial nerve

Kobayashi et al. (2022) [73]	Pfizer-BioNTech	NA	F/46	AE	Acute-onset diplopia	(i) Lesion on the dorsal pons across the midline

Rastogi et al. (2022) [74]	ChAdOx1(AstraZeneca) and mRNA-1273 (Moderna)	NA	F/59	AE	Unsteady gait, incoordination, dizziness, binocular diplopia, perioral paresthesia, right hand numbness, and lethargy	(i) CE T1-w showed multiple focal, poorly defined regions of contrast enhancement in the cerebral cortex, deep grey matter, brainstem, and cerebellum

Vences et al. (2022) [75]	Pfizer-BioNTech	DM and HTN	M/72	AE	Nausea, vomiting, malaise, headache, fever, confusion, aggressiveness, and gait alterations	(i) After first dose: T2-w hyperintense lesions at the bilateral straight frontal gyri, left cingulate, and insula
						(ii) After second dose: T2-w FLAIR lower-volume lesions in the bilateral frontal lobes compared to that in the previous MRI and new hyperintense lesions, predominantly in the left temporal region

Vences et al. (2022) [76]	BIBP-CorV (Sinopharm)	NA	F/33	AE	Headache, the sensation of thermal rise, conciliation insomnia, and transitory episodes of environment disconnection	(i) T2-w FLAIR shows small hyperintense non-specific demyelinating lesions at the periventricular region, internal capsule, and bilateral subcortical areas
						(ii) Second MRI: in the T2-w FLAIR sequence shows well-defined focal hyperintense lesions at the bilateral caudate nucleus and demyelinating lesions at the bilateral subcortical areas

Zhang et al. (2023) [77]	BIBP-CorV (Sinopharm)	NA	M/29	AE	Headaches, dizziness, muscle soreness, weakness, psychomotor agitation, intermittent confusion, intractable hiccups, and decreased appetite	(i) Abnormal signals in the splenium of the corpus callosum

Huang et al. (2022) [78]	ChAdOx1(AstraZeneca)	NA	F/38	Autoimmune encephalopathy	Acute-onset amnesia, language disturbance, and seizure	(i) DWI-MRI: a subacute infarction at the right internal capsule and irregular vascular contour
						(ii) MRV: irregularity of vascular contour

Youn and Yang (2021) [79]	Pfizer-BioNTech	NA	M/22	CLOCCs	Febrile sensation and headache around the eyes and forehead	(i) An oval-shaped restricted diffusion in the corpus callosum with low ADC values and lack of contrast mediated enhancement

Poussaint et al. (2021) [80]	Pfizer-BioNTech	Lyme disease	M/12	CLOCCs	Severe headache and visual hallucinations	(ii) The splenium of the corpus callosum revealed T2-w prolongation and lower diffusivity

Procaccini et al. (2022) [81]	Pfizer-BioNTech	NA	F/51	CLOCCs	Fever, weakness, headache, palpitations, malaise, and AMS	(i) Single oval-shaped lesion located in the splenium of the corpus callosum, hyperintense signal on T2-w and FLAIR, and restricted diffusivity on DWI
						(ii) The first follow-up MRI, after 17 days: a complete resolution of the lesion in the splenium of the corpus callosum and several small round-shaped WM hyperintensities on T2-w and FLAIR
						(iii) At the second follow-up MRI after 67 days: brain findings remained unchanged

Ohara et al. (2022) [82]	Pfizer-BioNTech	(1) NA	(1) M/23	CLOCCs	(1) Nausea, mild headache, and fever	1 and 2 brain MRI:
		(2) Mild mental retardation and depression	(2) F/33		(2) Disturbance and headache	(i) Restricted diffusion in the splenium with low ADC values, and FLAIR: a high signal intensity lesion at the midline of the splenium of the corpus callosum

Blauenfeldt et al. (2021) [83]	ChAdOx1 (AstraZeneca)	Hashimoto's thyroiditis and HTN	F/60	Acute ischemic stroke	Headache and persistent abdominal pain	(i) DWI: completed infarction in the entire area supplied by the right MCA
						(ii) MRA: occlusion of the right internal carotid artery

Elaidouni et al. (2022) [84]	BIBP-CorV (Sinopharm)	NA	M/36	Acute ischemic stroke	Numbness in his left arm and legs with headaches, asymmetry of the face, and disturbance of consciousness	(i) An extensive stroke ischemic in the superficial and deep right parietal territory with the onset of hemorrhagic rearrangement of the right basal ganglia

Rattanawong et al. (2021) [85]	Sinovac-CoronaVac	NA	F/24	Acute prolonged motor aura resembling ischemic stroke	Visual disturbance, numbness and weakness of the left arm, and tingling sensation over the fingers in the left hand	(i) MRA: mild irregularity of the left pericallosal artery

Suwanwela et al. (2022) [86]	Sinovac-CoronaVac	Migraine	F/24	Prolonged migraine aura resembling ischemic stroke	Tingling progress to numbness at left arm and leg, pulsatile headache in the left temporal	(i) Mild irregularities of the pericallosal branch of the intracranial artery

Yoshida et al. (2021) [87]	Pfizer-BioNTech	Atrial fibrillation and osteoarthritis	F/83	Cardioembolic stroke	Left hemiplegia and left hemispatial neglect	Brain MRI after first dose:
						(i) DWI: acute infarction in the left insular cortex and corona radiata. T2^∗^-w: susceptibility vessel signs are seen in the left middle MCA. MRA: the left MCA is occluded at the proximal M1 segment.
						Brain MRI after second dose:
						(ii) DWI shows acute infarction in the right insular cortex, caudate, and corona radiate
						(iii) T2^∗^-w: susceptibility vessel sign in the right MCA
						(iv) MRA: the right MCA is occluded at the proximal M1 segment

Fotiadou et al. (2022) [88]	Pfizer-BioNTech	NA	M/59	Spinal cord ischemia	Right lower limb weakness accompanied by abdominal pain and progressing to paraplegia	(i) T2-w hyperintensities extending from T6 level to conus medullaris and bilaterally symmetric circular high signal foci in the anterior horn cells of the spinal cord

Ancau et al. (2022) [89]	ChAdOx1 (AstraZeneca)	(1) Hypothyroidism and polymyalgia rheumatica	(1) M/61	AHEM	(1) Fever, headache, apathy, and generalized seizure	(1) FLAIR hyperintense lesions with hemorrhagic involvement of the basal ganglia on both sides of the brain
		(2) NA	(2) F/25		(2) Cephalgia, thoracic back pain, mild weakness, and ascending numbness in the legs	(2) A longitudinal edema with modest contrast enhancement and isolated central hemorrhage in thoracic spinal cord. WM abnormalities in both hemispheres, as well as localized amplification in the WM
		(3) NA	(3) F/55		(3) Developed progressive nausea, dizziness, and meningism	(3) In the right occipital and left frontobasal regions, many FLAIR hyperintense and hemorrhaging lesions were seen in the right parietal and temporal areas

Kits et al. (2022) [90]	Pfizer-BioNTech	Rheumatoid arthritis	M/53	AHEM	Confusion, unconsciousness, agitation, and snoring	(i) Multiple cortical and subcortical lesions with high T2-w and FLAIR signal and ubiquitous petechial hemorrhages
						(ii) Repeated MRI: development of widespread lesions in cortical GM, thalami, basal ganglia, corpus callosum, brainstem, and cerebellum. Multiple lesions, mostly in the GM, in the cervical and thoracic medulla.
						(iii) After three weeks, cortical laminar necrosis, a decrease in brain swelling, and regional encephalomalacia appeared in addition to increasing bilateral confluent lesions with increased signal in FLAIR and DWI, possibly representing delayed demyelination

Finsterer and Korn (2021) [91, 92]	mRNA-based SARS-CoV-2	MI, HTN, HLP, and nephrolithiasis	M/52	Intracerebral bleeding	Sudden-onset difficulties with reading and speaking (aphasia)	(i) T2-w and SWI: intracerebral bleeding in the left temporal lobe

Elnahry et al. (2021) [93]	ChAdOx1 (AstraZeneca)	NA	F/32	Optic neuropathy	Blurring of vision in the left eye, superior field loss, and pain with eye movement	(i) Brain, orbit, and spine MRI: significant enhancement of left optic nerve
Bagella et al. (2021) [94]	ChAdOx1 (AstraZeneca)	NA	M/49	Inflammatory demyelinating polyneuropathy	Asymmetric bilateral facial weakness and paresthesias in the tongue and face	(i) Enhancement of the facial nerves and of the cauda equina and lower thoracic nerve roots

Abbott et al. (2022) [95]	ChAdOx1 (AstraZeneca)	NA	(1) F/68	Acute small fiber neuropathy	(1) Dysesthesias and altered temperature sensation on hands and feet extending proximally	(1) Multiple perineural cysts attached to bilateral L5‐S2 nerve roots deemed incidental. Cystic dilatation of the distal spinal cord central canal.
			(2) F/55		(2) Bilateral neuropathic pain and paresthesias in the hands and feet. Cheeks, nose, and tongue numbness and paresthesias.	(2) Prominent central canal within the spinal cord from C4/C5 to mid T1-w vertebral level

Shalabi et al. (2022) [96]	Pfizer-BioNTech	NA	M/41	Unilateral multiple cranial neuropathy	Dysphagia, hoarseness, right side hearing loss, and diplopia	(i) Enhancement in the right auditory canal, which involved the VIIth and VIIIth CNs

Manea et al. (2021) [97]	Pfizer-BioNTech	NA	M/29	Cranial nerve palsies	Left oculomotor, abducens, diplopia, trigeminal, facial and Bell's palsies	(i) Diffuse gadolinium enhancement in the intra-canalicular and labyrinthic portion of the left facial nerve, intra-cisternal course of the left trigeminal, and oculomotor nerve

Cellina et al. (2021) [98]	mRNA-1273 (Moderna)	NA	F/36	Bell's palsy	Deep left latero-cervical pain and stiffness, followed by the sensation of not holding liquids properly in the mouth	(i) Facial and vestibulocochlear nerves entering the internal auditory meatus with regular morphology and thickness
						(ii) CE T1-w: enhancement of the distal intra-canalicular and labyrinthine segments of the left VII nerve

Correa et al. (2021) [99]	ChAdOx1 (AstraZeneca)	(1) HTN	(1) M/64	(1) Ischemic stroke	(1) Right superior and inferior limbs paresia	(1) Acute ischemic stroke in the left basal ganglia region, with hyperintense signal on FLAIR, associated with restricted diffusion, on DWI, with a reduced signal on ADC
		(2) NA	(2) M/42	(2) Facial nerve palsy	(2) Pain in the left ear, left facial muscles weakness, paresis of the left forehead's muscles, left lagophthalmos and labial hypomobility, and left peripheral facial nerve palsy	(2) Gadolinium enhancement in the canalicular and labyrinthine portions of the left facial nerve and in the left geniculate ganglion
		(3) NA	(3) M/65	(3) Myelitis	(3) Tetraparesia	(3) Cervical spine MRI shows a hyperintense lesion on STIR and on T2-w extending from C4 to C6 level, lateralized to the left portion of the spinal cord

Kulsirichawaroj et al. (2022) [100]	Pfizer-BioNTech	NA	F/16	Polyneuritis cranialis	Numbness and drooping on the right side of her face	(i) CE T1-w: abnormal enhancement of right cranial nerve VII at the canalicular and labyrinthine segments and the genu

Kizawa and Iwasaki (2022) [101]	Pfizer-BioNTech	HTN, HLP, and osteoarthritis	F/75	Amyloid *β*-related angiitis of the CNS	Frontal headache, depression, aphasia, apraxia, and a gait disturbance	(i) DWI: abnormal hyperintensity, suggesting cerebral infarctions in the left parietal and occipital lobes

Mahajan et al. (2023) [102]	(1) ChAdOx1 (AstraZeneca)	(1) NA	(1) M/38	Immune-mediated demyelination of the CNS	(1) Bilateral lower limbs with sensory loss and bladder disturbance	(1) Bilaterally symmetrical T2-w and FLAIR hyperintensities within the corticospinal tracts, involving the posterior internal capsules and corona radiate. Bilaterally symmetrical, long segment, T2-w hyperintensities in the spinal cord tracts involving the dorsal columns and bilateral lateral corticospinal tracts, extending from the cervicomedullary junction till the conus. Bilateral trigeminal nerve nuclei and intraparenchymal trajectory of trigeminal nerves also showed T2-w FLAIR hyperintensities.
	(2) COVAXIN	(2) Hypothyroidism	(2) M/50		(2) Tingling sensation in both feet, which progressed to involve the legs, and difficulty in walking	(2) Bilaterally symmetrical hyperintensities within the corticospinal tracts, involving the posterior internal capsules and corona radiata, and medullary pyramids, with long segment T2-w hyperintensities in the spinal cord tracts involving the dorsal columns and bilateral lateral columns, extending from the cervicomedullary junction till the conus. The bilateral intraparenchymal trajectory of trigeminal nerves and trigeminal nerve nuclei also showed T2-w FLAIR hyperintensities
	(3) NA	(3) NA	(3) M/38		(3) Progressive symmetric quadriparesis involving the lower limbs first and then progressing to the bilateral upper limbs	(3) Bilaterally symmetrical hyperintensities in the posterior limbs of the internal capsule, the bilateral intraparenchymal pontine trigeminal nerves, and the bilateral lateral columns and the dorsal columns of the spinal cord

Garg et al. (2022) [103]	ChAdOx1 (AstraZeneca)	HTN	F/56	Tumefactive demyelinating brain lesion	Weakness of the right upper and lower limbs	(i) T2-w FLAIR hyperintensities involving the WM of the left parietal lobe and extending into the body of the corpus callosum

Shiraishi et al. (2022) [104]	mRNA-1273 (Moderna)	Enlargement of the left testicle leads to testicular seminoma	M/32	Paraneoplastic tumefactive demyelination	Right-sided motor and sensory impairment	(i) High-intensity lesion in the left internal capsule then the brain lesion had enlarged and progressed to a tumefactive lesion

Roncati and Manenti (2022) [105]	Vaxzevria (formerly COVID-19 Vaccine AstraZeneca)	NA	F/28	Pituitary apoplexy	Long-lasting tension-type headache and menstrual changes	(i) Signal alteration (related to a hemorrhagic event) detected in the right half of the sella turcica
						(ii) The gadolinium CE MRI: pituitary apoplexy presence
Gogu et al. (2022) [106]	Ad26.COV2.S (Jcovden)	DM	M/45	Tolosa–Hunt syndrome	Fever, headache, and respiratory symptoms	(i) T2-w and FLAIR: perineural tissue extending into the left cavernous sinus; hypersignal in the left temporal lobe, the hippocampal, and para hippocampal
						(ii) CE T1-w: an inflammatory process involving the left cavernous sinus and orbital apex with perineural enhancement surrounding the left optic nerve sheath. MRV: thrombus in the cavernous sinus.

Santilli et al. (2022) [107]	(1) Pfizer-BioNTech	NA	(1) F/14	Multi-system inflammatory syndrome with overlapping neurological involvement	(1) Episode of unresponsiveness to stimuli, catatonia, and inability to move followed by agitation and confusion (2) Agitation and headache, followed by drowsiness	(i) T2-w hyperintensity in the splenium of the corpus callosum with restricted diffusion
	(2) mRNA-SARS-CoV2		(2) M/9			

Li et al. (2022) [108]	Sinovac-CoronaVac	Received the third injection of COVID-19 vaccine when the cold symptoms were not completely gone	M/70	Cervical *Staphylococcus aureus* infection	Severe neck and shoulder pain, accompanied by numbness and fatigue in the limbs	(i) Abnormal signals in the spinal cord at the level of C3 to C6 vertebrae, cervical vertebra infection, resulting in cervical abscess compression on the cervical spinal cord

Zhang et al. (2023) [109]	BIBP-CorV (Sinopharm)	Eczema and renal calculus	M/39	Charcot–Marie–Tooth disease type 1 (CMTX1)	Aphasia, asyndesis, dysphagia, and dyspnea	(i) Bilateral, symmetrical, and restricted diffusion, with T2-w hyperintensities in the corpus callosum and supratentorial WM

## Data Availability

The data used to support the findings of this study are included within the article. Any further inquiries should be forwarded to the corresponding author.

## Abbreviations

ADEM: Acute disseminated encephalomyelitis
AHEM: Acute hemorrhagic encephalomyelitis
ATM: Acute transverse myelitis
AEFI: Adverse events following immunization
AMS: Altered mental status
Anti-NMDAR: Anti-N-methyl-d-aspartate receptor
ADC: Apparent diffusion coefficient
AE: Autoimmune encephalitis
BPH: Benign prostatic hyperplasia
CNS: Central nervous system
CBF: Cerebral blood flow
CBV: Cerebral blood volume
CVST: Cerebral venous sinus thrombosis
CMTX1: Charcot–Marie–Tooth disease type 1
CE: Contrast-enhanced
CLOCCs: Cytotoxic lesions of the corpus callosum
DM: Diabetes mellitus
DWI: Diffusion-weighted imaging
DIR: Double inversion recovery
F: Female
FLAIR: Fluid-attenuated inversion recovery
GM: Grey matter
GBS: Guillain–Barré syndrome
HLP: Hyperlipidemia
HTN: Hypertension
LTEM: Longitudinally extensive transverse myelitis
MRA: Magnetic resonance angiography
MRI: Magnetic resonance imaging
MRV: Magnetic resonance venography
MDD: Major depressive disorder
M: Male
MCA: Middle cerebral artery
MS: Multiple sclerosis
MOG: Myelin oligodendrocyte glycoprotein
MI: Myocardial infarction
NMOSD: Neuromyelitis optica spectrum disorder
NA: Not applicable
PWI: Perfusion-weighted imaging
PRISMA: Preferred Reporting Items for Systematic Reviews and Meta-Analyses
SARS-CoV-2: Severe acute respiratory syndrome coronavirus 2
STIR: Short tau inversion recovery
SWI: Susceptibility-weighted imaging
T1-w: T1-weighted
T2-w: T2-weighted
TM: Transverse myelitis
VITT: Vaccine-induced immune thrombotic thrombocytopenia
WM: White matter
WHO: World Health Organization.
